# Tailoring Cellular Function: The Contribution of the Nucleus in Mechanotransduction

**DOI:** 10.3389/fbioe.2020.596746

**Published:** 2021-01-08

**Authors:** Fabrizio A. Pennacchio, Paulina Nastały, Alessandro Poli, Paolo Maiuri

**Affiliations:** ^1^FIRC (Italian Foundation for Cancer Research) Institute of Molecular Oncology (IFOM), Milan, Italy; ^2^Laboratory of Translational Oncology, Institute of Medical Biotechnology and Experimental Oncology, Medical University of Gdańsk, Gdańsk, Poland

**Keywords:** mechanosensing, nuclear envelope, LINC complex, cell nucleus, mechanotransduction

## Abstract

Cells sense a variety of different mechanochemical stimuli and promptly react to such signals by reshaping their morphology and adapting their structural organization and tensional state. Cell reactions to mechanical stimuli arising from the local microenvironment, mechanotransduction, play a crucial role in many cellular functions in both physiological and pathological conditions. To decipher this complex process, several studies have been undertaken to develop engineered materials and devices as tools to properly control cell mechanical state and evaluate cellular responses. Recent reports highlight how the nucleus serves as an important mechanosensor organelle and governs cell mechanoresponse. In this review, we will introduce the basic mechanisms linking cytoskeleton organization to the nucleus and how this reacts to mechanical properties of the cell microenvironment. We will also discuss how perturbations of nucleus–cytoskeleton connections, affecting mechanotransduction, influence health and disease. Moreover, we will present some of the main technological tools used to characterize and perturb the nuclear mechanical state.

## Introduction

In living systems, cells are continuously exposed to a complex pattern of chemical and physical stimuli coming from the functional features of the surrounding microenvironment. Cells sense and integrate such inputs thanks to a wide range of receptors that, by initiating specific signaling cascades and/or inducing a morphological cellular reshaping, then trigger a variety of intracellular events.

Mechanically, cells could be modeled as a hypothetical pre-stressed dynamic system, where the cytoskeleton organization balances forces between opposite structural elements (Wang et al., 2009). As a consequence, forces applied to cells are propagated, through cytoskeletal components, from the extracellular environment to the different cellular organelles. Among them, the nucleus is the stiffest one (Kirby and Lammerding, 2018) and can undergo significant shape deformations when mechanically perturbed, triggering multiple gene-expression patterns (Humphrey et al., 2014; Alisafaei et al., 2019). The ability of cells to convert microenvironment mechanical stimuli into biochemical cascades, mechanotransduction, is critical in several biological processes, such as migration, proliferation, differentiation, embryogenesis, and tissue homeostasis and repair (Humphrey et al., 2014). Interestingly, the nucleus is emerging as a fundamental player for cellular mechanosensing, and its capability to deform and properly react to external mechanical cues is probably strictly related to the functionality of its load-bearing elements (Navarro et al., 2016). Recent publications pointed out that the nucleus tensional state is a sensor of both cellular compression and stretching (Jiménez-delgado et al., 2020; Lomakin et al., 2020). In fact, when a cell is spatially constrained, the extent of nuclear deformation and the consequent stretching of the NE trigger remodeling of actomyosin cortex finally leading to increased cellular contractility (Lomakin et al., 2020). This, in turn, by inducing the opening of nuclear pores, controls the access to the nucleus of YAP (Elosegui-Artola et al., 2017), a well-characterized transcription factor regulating mechanotransduction (Dupont et al., 2011). The central role of the nucleus is also highlighted by the finding that nuclear structural defects, leading to impaired mechanotransduction, are associated with several pathologies. For example, laminopathies are rare genetic diseases caused by mutations of genes encoding the NE proteins such as Lamin A/C (Lamin), Emerin (EMD), and Nesprins (SYNE1/2). These pathologies, which include muscular dystrophies like EDMD, lipodystrophy syndromes, and progeroid syndromes, often present impaired nucleus–cytoskeleton force transmission, which affects the capacity of cells to correctly respond to mechanical stimuli (Lammerding et al., 2004). Moreover, altered nuclear shape and size have been used for many years as a hallmark of cancer (Uhler and Shivashankar, 2018). Cancer cell nuclei often present lower nuclear stiffness and higher deformability, which have been hypothesized to directly contribute to the cellular invasion capability and then to metastasis dissemination (Denais and Lammerding, 2014).

The growing evidence supporting the crucial role of the nucleus in mechanosensing has promoted the development of experimental tools to detect cell responses to controlled and finely tuned nuclear stresses and allow functional dissection of the mechanotransduction processes (Ingber, 2018). This represents a multidisciplinary field, which combines cutting-edge technologies in complementary research areas, such as materials science, cell and molecular biology, and advanced imaging. The use of engineered materials and platforms provides unparalleled opportunities of mechanical stimulation by modulating cellular tensional state (Song et al., 2020). Here, micro- and nano-fabrication techniques, chemical patterning, and material chemistry allow in fact the fine-tuning of cell/substrate interaction. The induced cell responses can be then analyzed at the molecular, morphological, or ultrastructural level to unravel the molecular and genetical processes underpinning mechano-mediated function regulation (Uhler and Shivashankar, 2017).

In this review, we provide an overview about how the nucleus contributes to cell mechanotransduction in both normal and pathological conditions, highlighting state-of-the-art methodologies developed to alter and analyze nuclear mechanics.

## Nuclear Structure Components and Their Role in Mechanosensing

**FIGURE 1:**
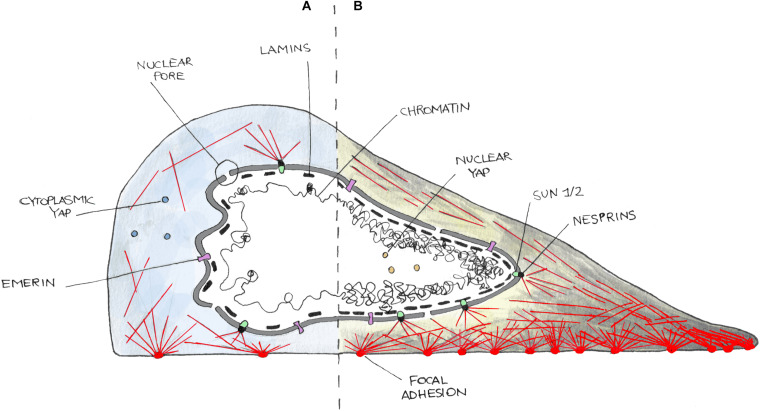
Cartoon representing the nucleus–cytoskeleton–extracellular matrix connections in cells in a low contractility state (i.e., weak adhesions) **(A)** or in high contractility one (i.e., strong adhesions) **(B)**. On **(A)**, cells poorly adhere to the substrate and develop few and small focal adhesions and thin stress fibers (red lines). Cells in this state are characterized by cytoplasmic localization of YAP and often present nuclear envelope (NE) invaginations. In **(B)**, cells well adhere and spread on the substrate and form more and bigger focal adhesions as well as thick stress fibers. This induces a higher internal tension then transmitted to the NE, leading to nuclear invagination disappearance and nuclear translocation of YAP. NE is here represented as a double bilayer, supported by the Lamin meshwork in the nucleoplasm, and connected to the cytoskeleton by the LINC complex made up of SUN (SUN1/2) and KASH (Nesprins) domain containing enzymes, and Emerin.

The nucleus can be structurally and functionally divided into two compartments: the nuclear interior (nucleoplasm) and the NE (Wilson and Berk, 2010) (Figure 1). The nuclear interior is mostly aqueous, and it is composed of functional substructures that can be affected by mechanical stress including nucleoli (Shim et al., 2019) and Cajal bodies (Poh et al., 2012). The nucleoplasm is enclosed by the NE, which is composed of two lipid bilayers, the INM and ONM that is contiguous with the ER (Schwarz and Blower, 2016; Ungricht and Kutay, 2017). These two leaflets are separated by a luminal space of 30–50 nm called PNS or lumen. The principal function of the NE is to act as a barrier for genetic material protection, allowing faithful replication and regulated transcription. On the NE, the NPCs are responsible for the bidirectional transport of proteins and ions inside and outside the nucleus (Knockenhauer and Schwartz, 2016). Despite its structural role in separating and organizing the genome, the NE forms a dynamic and adaptable membrane that anchors proteins involved in transmission of mechanical signals. Indeed, the NE is connected with both the inner and outer sides of the nucleus. In fact, the INM is interconnected to chromatin through a meshwork of filaments, type A and type B lamins (Bergo et al., 2004). These proteins have pivotal roles in gene expression and chromatin organization and are involved in the maintenance of proper nuclear architecture, acting together with the LINC complex in order to regulate nuclear stiffness, viscoelastic behavior, and response to mechanical stimuli (Alam et al., 2016; Janin et al., 2017).

### LINC Complex

Cells interact with the ECM through integrins (Kechagia and Ivaska, 2019). Cell/substrate interaction is then stabilized thanks to the formation of focal adhesions, multi-protein clusters generated around the original site of integrin–ECM engagement, which include Talin, Vinculin, and/or Paxillin, and other signaling molecules such as focal adhesion kinases (Parsons et al., 2010). Upon maturation, focal adhesions can transmit external mechanical stimuli to the cytoskeleton, which hereafter transfer it to the LINC complex (Figure 1). This, connecting the nucleus to the cytoskeleton, finally accomplishes the physical link between the microenvironment and the nucleus, enabling the entire cell to act as a mechanically coupled system (Lombardi et al., 2011; Chang et al., 2015). At the inner site of the LINC complex, there are the SUN domain-containing proteins (named after homologous sequences shared by *Schizosaccharomyces pombe* proteins: Sad1p, UNC-84). They span the INM and provide an anchorage between the inner NE and the lamin meshwork in the nucleoplasm (Lombardi et al., 2011). SUN domain-containing proteins (SUN 1 and 2) have been shown to play critical but redundant functions during development (Lei et al., 2009; Yu et al., 2011); however, recent studies suggest that they play separate roles ([Bibr B141][Bibr B170]). SUN proteins interact with other elements of the LINC complex, the Nesprins. Nesprins contain a conserved C-terminal KASH domain, present at the ONM (Zhang et al., 2001; Lombardi et al., 2011). SUN and KASH domain proteins interact at the PNS. Numerous Nesprins isozymes exist in mammalian cells, with redundant and non-redundant functions. For example, both Nesprin-1 and -2 are present in different isoforms due to alternative initiation/termination of transcription and/or alternative splicing of *SYNE1* and *SYNE2* genes. These isoforms have a highly tissue-dependent expression and exhibit different subcellular locations (Rajgor et al., 2012; Duong et al., 2014) that could possibly tailor the mechanical response upon different stimuli in various environments. For example, Nesprin-1α isoform is required for the recruitment of several centrosomal proteins to the NE during skeletal muscle formation (Gimpel et al., 2017), whereas Nesprin-2 accumulates at the front of the nucleus during confined cell migration (Davidson et al., 2020). Giant isoforms of Nesprin-1 and -2 possess an N-terminus CH domain, through which they bind F-Actin (Rajgor and Shanahan, 2013). Despite the absence of a CH domain, Nesprin-3 and -4 can connect with cytoskeleton, too. In particular, Nesprin-3 links intermediate filaments through a Plectin-binding domain and is essential in fluid shear-induced polarization of the centrosome and directional migration of human aortic endothelial cells (Wilhelmsen et al., 2005; Morgan et al., 2011). Finally, Nesprin-4 can indirectly bind microtubules and is involved in kinesin-mediated cell polarization (Roux et al., 2009).

### LEM-Domain Containing Proteins

LEM domain-containing proteins bind Lamins and are known to be involved in the tethering of repressive chromatin at the nuclear periphery (Berk et al., 2013; Barton et al., 2015). Recent evidence suggests that they can form hubs within the nuclear lamina that integrate external signals. Emerin was shown to interact with both nuclear and cytoplasmic actin (Lattanzi et al., 2003), and it is known for its actin-capping properties (Holaska et al., 2004) (Figure 1). It was reported to be enriched at the ONM under cyclic strain stress (Le et al., 2016), whereas Emerin-deficient cells showed impaired expression of mechanosensitive genes in response to strain (Lammerding et al., 2005). Emerin has also been recently shown to be associated with nuclear stiffening and to be involved in maintaining nuclear front–rear polarity (Nastały et al., 2020). Mutations in Emerin-encoding gene (*EMD*) cause X-linked EDMD that is associated with progressive muscle wasting and weakness followed by cardiac disease with conduction defects and arrhythmias (Helbling-Leclerc et al., 2002).

### Lamins

The nucleoskeleton is mainly composed of intermediate type V filaments that can be separated into A-type and B-type lamins (Figure 1). Lamins B1 and B2 are ubiquitously expressed products of independent genes (*LMNB1* and *LMNB2*, respectively), essentials for early development (Bergo et al., 2004; Coffinier et al., 2010). In contrast, the A-type lamins including Lamins A and C (alternatively spliced isoforms of the *Lamin* gene) are expressed mainly in differentiated cells, and generally later in development (Rober et al., 1989; Sullivan et al., 1999). They are located in the INM and contribute to nuclear stiffness, integrity, and are involved in nuclear mechanotransduction processes. Lamin A plays a critical role in localizing other NE components including Lamin C (Vaughan et al., 2001), Nesprin-2 (Libotte et al., 2005), or Emerin (Vaughan et al., 2001). Lamin A/C deficiency causes defective nuclear mechanics and mechanotransduction (Lammerding et al., 2004). Moreover, defects of the nuclear lamina are mechanistically associated with the accumulation of DNA damage, DNA repair machinery activation, and cell death, features identified in all the laminopathies (Earle et al., 2020). Here, Lamin-deficient cells have been found to be more sensitive to cytoskeletal forces developed during skeletal muscle cell migration and maturation, which caused the rupture of the NE and the exposure of the genetic content to the cytoplasmic DNA nucleases (Earle et al., 2020). Mutations in the Lamin gene are the main cause of disorders named laminopathies, which are characterized by the presence of cells with irregular shaped nuclei. Although at least 15 different types of laminopathies exist, which makes them the highest number of diseases related to a single gene mutation (Schreiber and Kennedy, 2013), it is still unclear how various Lamin mutations cause different, often system-specific, disease phenotypes leading to various muscular dystrophies, lipodystrophies, progeroid syndromes, and many more (Worman and Bonne, 2007; Worman, 2012).

### Nuclear Pore Complex

The NPC is a complex of more than 70 different proteins that spans over the NE and enables protein and RNA trafficking between the nucleus and the cytoplasm (Knockenhauer and Schwartz, 2016) (Figure 1). Recently, it has been shown that mechanical forces may significantly affect the basket conformation of NPC via the Nup153–SUN1 interaction (Li and Noegel, 2015). Moreover, exposure to stiff substrates can also influence nuclear pore stretching, reducing their mechanical resistance to molecular transport and, in consequence, leading to increased YAP nuclear import (Elosegui-Artola et al., 2017).

## Impaired Mechanosensing in Cancer

A perturbed nuclear structure corresponds to an altered spatial organization of the genome, a key regulator of gene expression, whose alteration has already been shown to drive tumor transformation in human cellular models of glioma and leukemia (Flavahan et al., 2016; Hnisz et al., 2016). In addition, imposition to cells’ specific geometries affects both nuclear shape (Versaevel et al., 2012) and gene expression (Jain et al., 2013), supporting the idea that unexpected changes of cell shape might trigger a reorganization of the three-dimensional conformation of the genome. This could subsequently induce variations in gene expression patterns, potentially leading to the activation of proto-oncogenes or the silencing of tumor suppressor genes. As previously mentioned, nuclear morphology analysis is a well-established marker for cancer diagnosis since cancer cell nuclei are often characterized by abnormal size, irregular shapes (i.e., invaginations), and different mechanical properties compared to their normal counterpart. Moreover, alteration of nuclear stiffness, NE composition, and chromatin distribution have been reported in several tumors (Zink et al., 2004; Saarinen et al., 2015; Irianto et al., 2016; Reis-Sobreiro et al., 2018). Several studies have highlighted how mechanical interactions between cells and ECM affect tumor onset and evolution. A crucial milestone in the field was the discovery that cell stiffness, reflecting the stiffness of the surrounding microenvironment (Discher et al., 2005), could regulate both tumorigenesis and cancer cell proliferation through the activation of the transcription factor YAP (Dupont et al., 2011). Moreover, tissue mechanical properties have been linked to tumor progression (Mouw et al., 2014; Nam et al., 2019), metastasis formation (Liu et al., 2017; Rice et al., 2017), and dedifferentiation toward malignant phenotypes (Li et al., 2020). All these findings underlie a tight connection between mechanically induced cytoskeletal modifications and nuclear structural elements, whose properties then affect gene expression profile and cancer cell behavior. Interestingly, the majority of the genes encoding NE proteins do not show clear general mis-regulations or frequent mutations in tumors. However, upregulation of genes encoding Lamin B1 and B2 or the nucleoporin NUP210 is often found in cancer, while SYNE1 and SYNE2, genes encoding Nesprins, turn out to be almost always downregulated (De las Heras et al., 2013). Nuclear mechanical properties have a crucial role in metastasis dissemination as well, which cause about 80% of cancer-related deaths (Hamidi and Ivaska, 2018). During this process, cancer cells must acquire a migratory phenotype and then overcome the structural barrier imposed by the surrounding microenvironment (i.e., other cells, the hosting tissue, the blood vessels, and the tissue to invade). More specifically, metastatic cells can squeeze themselves in interstitial spaces of size below 2 μm, undergoing cytoskeletal rearrangements and severe nuclear deformations typically inaccessible for normal cells (Denais and Lammerding, 2014). In line with these data, low levels of Lamin A/C, often observed in gastric (Wu et al., 2009) and prostate cancer (Saarinen et al., 2015), are associated with nuclear softening facilitating nuclear deformation (Thiam et al., 2016). Interestingly, in colorectal cancer, instead, increase of Lamin A/C levels triggers cytoskeletal rearrangements linked to an enhanced cell motility and invasiveness (Saarinen et al., 2015). Moreover, defects of the nuclear lamina have been found to positively correlate with cytoskeletal-mediated nuclear ruptures during cancer cell migration, which have been hypothesized to affect the accumulation of genetic mutations leading to the acquisition of more malignant phenotypes (Vargas et al., 2012). Additionally, a recent study showed that mislocalization of Emerin, a structural protein of the NE, discriminated cancer from healthy tissues and correlated with disease progression in prostate cancer (Reis-Sobreiro et al., 2018). Emerin was also pointed as a mediator of nuclear shape stability, and in cancer, its destabilization promotes metastasis (Reis-Sobreiro et al., 2018).

## Methods to Alter and Measure Nuclear Mechanical State

**FIGURE 2:**
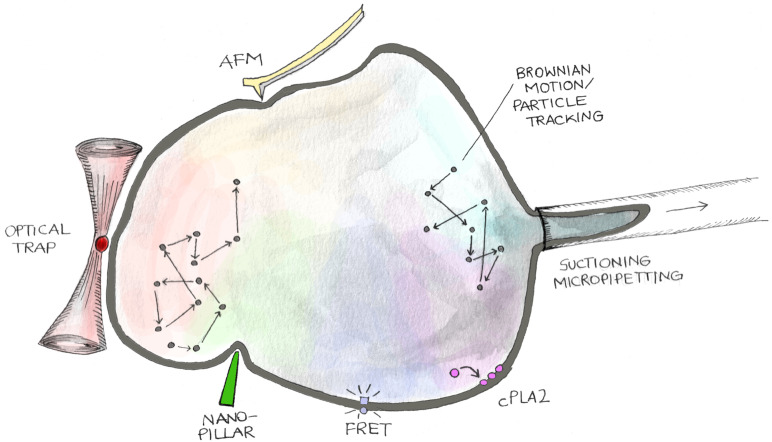
Methodologies to analyze mechanical features of cell nuclei. The cartoon shows different approaches to measure nuclear mechanical cues. Nuclear stiffness and viscosity can be measured by evaluating stress–strain curves generated by applying controlled mechanical perturbations through suctioning/micropipetting, atomic force microscopy (AFM), active–passive micro/nano-rheology (Brownian Motion/Particle Tracking), nano-pillar mediated nuclear deformation, and optical trap. On the other hand, tension exerted on the nuclear envelope can be quantified employing FRET probes genetically encoded into LINC proteins (Mini-Nesprin-2G), nucleoplasm/inner nuclear membrane translocation of cytoplasmic phospholipase A2 (cPLA2), and FliptR (fluorescent lipid tension reporter) membrane probes.

The numerous evidences highlighting nuclear contribution to mechano-regulation of cell behavior associated with the rapid growing of bioengineering and materials science prompted the development of strategies to both measure and specifically perturbate nuclear mechanics (Figure 2).

### Nucleus Mechanics Measurement

Nuclear mechanical properties (i.e., stiffness, elasticity, viscosity) could be characterized in several ways through different technologies (Wu et al., 2018). With the exception of passive rheology, nuclear mechanical features are generally measured analyzing the stress–strain curves generated through the application of controlled forces or deformations to the nucleus (Anselme et al., 2018) (Figure 2).

#### Micropipette Aspiration

One of the oldest and most reported techniques is micropipette aspiration (or suctioning), which has been employed for characterizing nuclear mechanics in both isolated nuclei or intact cells. Here, mechanical properties are evaluated by applying a controlled negative pression (i.e., aspiration) to the nucleus and measuring its deformation. Dahl et al. (2005), performing micropipette aspiration on isolated nuclei of TC7 cells (African green monkey kidney epithelium), demonstrated the distinct roles of chromatin and Lamin B in the viscoelastic nuclear response. Successively, Pajerowski et al., applying this technique to the entire cell body, discovered a positive correlation between nuclear stiffness and progression of cell differentiation. Moreover, they assessed how chromatin and Lamin differently regulate nuclear rheology and deformability (Pajerowski et al., 2007). Neelam et al. employed micropipette aspiration (to the whole cell) to investigate the structural elements involved in the maintenance of nuclear shape and position in homeostasis. They observed that elastic forces developed in response to mechanical perturbation and necessary to restore initial nuclear position and shape are mainly linked to the action of intermediate filaments network composed by vimentin, Lamin A/C, and SUN-domain proteins (Neelam et al., 2015).

#### Indentation

Another reported method to measure nuclear mechanics is indentation, which could be performed with dedicated micro-indentation systems or by AFM. In both cases, a deformation is imposed by indenting the nucleus with a tip placed to the extremity of a cantilever (Figure 2). The mechanical properties are then probed by measuring the cantilever deflection. The main difference between these two approaches is in the force application area (essentially the dimension of the tip), which ranges from several micrometers in the case of micro-indentation systems to few nanometers in the case of AFM, then leading to uniform or local nuclear compression, respectively (Anselme et al., 2018). Indentation has been performed on both isolated nuclei and intact cells and allowed evaluating different traits of nuclear mechanics. Schäpe et al. (2009) used AFM to measure the mechanical properties of isolated nuclei and they reported a positive correlation between Lamin A expression and nuclear stiffness. More recently, Wintner et al., combining data from indentation rheology and micropipetting aspiration, developed an elegant model to decipher the different contribution of chromatin and Lamins to nuclear viscoelasticity. They proposed that nuclear stiffness and chromatin condensation depend on both Lamin A and B1 expression, while nuclear viscosity is mainly determined by Lamin A (Wintner et al., 2020).

However, the use of these techniques presents some drawbacks mainly linked to technological limitation and to interpretation of the results. Indeed, the inability to directly access to the nucleus of adherent cells (i.e., non-isolated nuclei) limits the possibility to distinguish and properly evaluate the function of different cellular compartments in mechanotransduction processes. Moreover, physical models adopted to estimate mechanical properties from cantilever deflection measurement make assumptions/approximations that do not take into account the extremely complex and heterogeneous fluid-dynamic nature of the nucleus and, in general, of the cell (Krieg et al., 2019).

#### Particle Tracking

Particle tracking is a powerful technique to measure mechanical properties of different cellular compartments (Wirtz, 2009) (Figure 2). This method relies on the tracking of micro- and nano-sized objects injected into the cell. Rheological properties of the hosting cellular compartment are then extrapolated analyzing the objects’ trajectories. Basically, two different strategies could be approached: passive and active micro-rheology. In passive micro-rheology particles, trajectories are generated by spontaneous Brownian fluctuations and the MSD negatively correlates with local stiffness/viscosity. In active micro-rheology, instead, particle motion is triggered and controlled by external energetic sources such as light or magnetic fields. Here, cellular mechanical properties could be evaluated by both following particle trajectories in function of the applied force or by imposing controlled deformations (i.e., optical or magnetic tweezers). One of the first works analyzing nuclear mechanics through passive rheology was published in 2004 by Tseng et al. The authors separately evaluated the cytoplasmic and nuclear stiffness, recording higher mechanical properties in the latter. Moreover, within the nucleus, the elastic component was found to be predominant compared to the viscous one, leading to the hypothesis that nuclear structural coherence is mainly preserved by elastic mediated mechanisms (Tseng et al., 2004). Successively, Celedon et al. employed magnetic nanorods to infer viscoelastic properties of the nucleus in embryonic fibroblasts. By measuring nanorod rotations upon the application of a magnetic field, they highlighted the role of Lamin A/C in determining nuclear interior elasticity and viscosity (Celedon et al., 2011). Magnetic tweezers have also been used on isolated nuclei to assess nuclear mechanical properties independently from the possible activation of cell surface receptors. In these conditions, a nuclear stiffening was observed in response to force application (Guilluy et al., 2014). Recently, Wang et al. developed a multipole magnetic tweezers system able to finely control nanoparticle trajectories in 3D with a precision down to 0.4 μm. Interestingly, this innovative method allows the discovery of a spatial polarity of nuclear stiffness that correlates with the orientation of cytoskeleton actin filaments (Wang et al., 2019). Finally, Hanson et al. employed a substrate made of high-aspect-ratio quartz nanopillars to tune nuclear curvature and deformation in adherent cells. They analyze the different roles of NE composition, actin, and intermediate filaments in affecting nuclear deformation (Hanson et al., 2015).

#### FRET Biosensors

Many studies hinted to the analysis of membrane tension employ calibrated FRET-based tension biosensors, known as TSmod, genetically encoded into proteins involved in the response to mechanical stimuli (Grashoff et al., 2010) (Figure 2). The TSmod consists in a pair of fluorescent proteins (usually Cerulean and Venus like) acting as donor/acceptor for FRET analysis, linked by an elastic peptide that can elongate (low FRET) or shorten (high FRET) depending on the deformation imposed to the protein. This kind of sensor, once inserted in structural proteins such as Actin, could sense the different tensional status of the enzyme, allowing the study of forces at the cell–cell or cell–matrix levels (Conway et al., 2013; Cai et al., 2014; Gayrard and Borghi, 2016). Interestingly, this novel tool was extended to the study of forces exerted on the nucleus exploiting the NE components like Nesprins. Arsenovic et al. (2016) employed a chimera of giant Nesprin-2, named Mini_Nesprin-2G, which maintained the CH and KASH domains and behaved similarly to the original enzyme (Östlund et al., 2009). TSmode was inserted in the protein sequence and allowed the measurement of mechanical forces between actin cytoskeleton (CH domain) and nucleus (KASH domain). In particular, forces exerted on the NE became spatially different (apical vs basal) and increased in elongated fibroblasts. Moreover, cells seeded on different substrates and/or treated with compounds able to affect actin organization (Latrunculin) showed strong changes in the nuclear tensional state (Carley et al., 2020). However, few things must be considered. Mini_Nesprin2G lacks important domains present in the original protein, including FHOD1 and binding sites for microtubule motor proteins kinesin and dynein (Kutscheidt et al., 2014; Chang et al., 2015). All of these can contribute to the exertion of forces on the nucleus, so the Mini_Nesprin2G should be considered just as a tool to analyze forces. On the other hand, because many variants of the TSmod biosensor exist, characterized by different linkers, like the newest HP35 elastic peptide (Austen et al., 2015), or conformational derivatives of the two FRET probes (Meng and Sachs, 2012), they could potentially sense differently the nuclear tensional states. Finally, since Nesprins locate at the ONM and the mechanical model of how forces are transduced inside the nucleus is still debated, novel biosensors built on SUN1/SUN2 proteins are necessary.

#### FLIPPERS Probes

Another recently developed membrane tension sensor is the so-called FliptR (fluorescent lipid tension reporter), which specifically targets lipids in cell membranes and allows the analysis of different tensional states through FLIM (Fin et al., 2012; Dal Molin et al., 2015; Colom et al., 2018). In particular, FliptR derives from existing planarizable push–pull probes able to measure changes in lipid packing. In brief, lipid packing is defined as lipid acyl chain density: higher packing means more ordered acyl chains, while lower packing corresponds to more spaced acyl chains. So, the two dithienothiophene flippers composing FliptR are twisted and do not conjugate in non-confining conditions. On the other hand, upon external mechanical pressure on cell membranes that affect lipid organization, flippers planarize, conjugate, and change their fluorescence lifetime. FliptR has been exploited to analyze membrane tension in different cellular organelles, including the nucleus. Recently, Nava et al. (2020) showed through FLIM imaging of the FliptR reporter that tension on the nuclear membrane is reduced after 30 min of 40% stretch using a custom-built uniaxial cell stretcher (Faust et al., 2011; Noethel et al., 2018).

#### Activation of Cytoplasmic Phospholipase A2 (cPLA2)

The activation of cPLA2 has been successfully employed to study forces exerted on cell nucleus (Figure 2) (Enyedi et al., 2016). Phospholipases are enzymes able to hydrolyze PL into fatty acids and other lipophilic derivatives. PL are cell membrane components characterized by a hydrophilic head phosphate group and two fatty acid chains (often arachidonic and stearic acid) (Divecha and Irvine, 1995; Poli et al., 2019). PL are also involved in many signal transduction pathways. Four main classes of phospholipases exist: PLA, B, C, and D (Fiume et al., 2019). Phospholipases A cleave the *sn*-1 (PLA1) or the *sn*-2 (PLA2) PL acyl chains (Richmond and Smith, 2011; Gottardi and Luxton, 2020). Cytosolic Phospholipase A2 (cPLA2) is characterized by a Ca^2+^-dependent lipid binding C2 domain and a catalytic α/β hydrolase domain. Tissue damage activates cPLA2, triggering arachidonic acid release, which, in turn, is oxidized to pro-inflammatory eicosanoids by the 5-LOX at the NE (Enyedi et al., 2016). Studies performed both *in vivo* and *in vitro* showed that upon NE stretching and swelling, i.e., increased tension, the nucleoplasmic inactive portion of cPLA2 translocates to the inner NE where it triggers arachidonic acid release (Enyedi et al., 2016; Jiménez-delgado et al., 2020; Lomakin et al., 2020). This event is partially due to increased Ca^2+^ levels in the cytoplasm. The behavior of this specific phospholipase could then be considered as a novel tool to understand the tensional state of NE.

### Nucleus Mechanical Perturbation

To study the molecular mechanism underlying mechanotransduction, it is crucial to finely control cellular and then nuclear mechanical state. The nucleus can be mechanically stimulated either through the application of external forces or by tuning cell adhesion in order to impose a specific cytoskeletal organization (Figure 2). In tissues, cells are continuously subjected to a variety of mechanical forces such as compression, stretching, and shear stresses, which are determinant in the regulation of several physiological and pathological processes. Then, *in vitro* recapitulation of such stimuli represents a powerful approach to obtain a more comprehensive picture of the factors determining *in vivo* cellular behavior. Hereafter, we will discuss some of the most recognized methodological approaches in the field.

#### Compression

**FIGURE 3:**
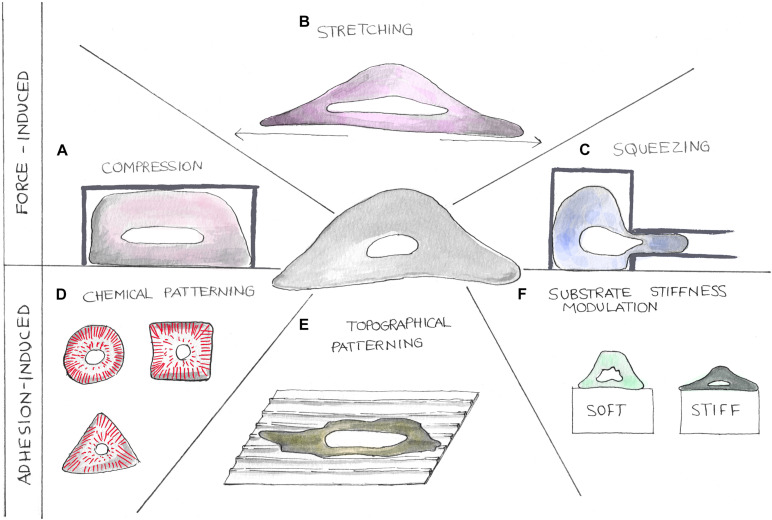
Cartoons showing different strategies to perturb the nuclear tensional state. Mechanical properties of cell nuclei can be altered using different methodologies. Forces can be applied on cells by compression, stretching, and squeezing devices **(A–C)**. Nuclear tensional state can also be indirectly controlled by altering cellular adhesion processes **(D–F)**. Here, chemical patterning **(D)**, topographical patterning **(E)**, and substrate stiffness modulation **(F)** can be exploited to alter cellular shape causing cytoskeletal component reorganization.

Three-dimensional cell confinement platforms are the most common tools to perform cell compression, a condition to mimic *in vitro* the extremely compact *in vivo* cellular microenvironment (Pampaloni et al., 2007) (Figure 3A). Liu et al., by confining cells with a micropillar-based system, discovered the mechano-mediated MAT, a possible mechanism to foster cancer cell dissemination. They demonstrated that, together with adhesion modulation, micrometric variations of cellular confinement (i.e., 2 μm of difference) could trigger MAT (Berre et al., 2015). 3D cell confinement has also been shown to affect cell proliferation. In fact, Lancaster et al., compressing cells with polymers of different stiffness, highlighted the role played by actin cortex in mitotic progression and spindle morphogenesis, whose failure is associated to the arise of cell division defects (Berre et al., 2013). Using a similar approach, Matthews et al. confined MCF10A cells to recapitulate the mechanical confinement experienced by cells in crowded tumors. They found that the expression of the Ras^V12^ oncogene promotes tumor progression by altering cell mechanics and then allowing cell division also under severe mechanical confinement, while normal cells displayed mitotic arrest or chromosome segregation errors (Matthews et al., 2020). Using polyacrylamide gels of different stiffness to confine cells (i.e., encapsulated in Matrigel), Li et al. (2020) showed that compression, together with osmotic pressure and substrate stiffness, leads to adipocyte reprogramming into multipotent cell lineage and enhances human mammary adenocarcinoma cell proliferation.

#### Stretching

In organs like heart or lung, cells are continuously subjected to stretching stimuli whose extension is known to impact upon the functioning of the tissue itself (Figure 3B). By plating cells on a silicone elastic membrane and then stretching it (20% of sustained stretch), Liao et al. studied the effects of abnormal stretching on cardiomyocyte and cardiac fibroblast viability, linking this mechanical perturbation to mitochondrial-dependent apoptotic pathways. Indeed, they found that mechanical stimulation triggers apoptosis mediated by mitochondria in cardiomyocytes and an upregulation of the cell cycle inhibitor p21 (Abbas and Dutta, 2009) together with a downregulation of Cyclin B1 in fibroblasts (Gavet and Pines, 2010). Coupling these findings with the most recent publications, we can speculate that thanks to the tight connection existing between the cytoskeleton and the nucleus, the tension exerted on the cell by the stretching is then propagated to the NE triggering the arrest of the cell cycle in G2/M (Liao et al., 2004). Cyclic stretching has also been associated with cellular spreading and growth. With a similar approach, Cui et al. (2015) have found that by cyclically stretching cells on soft substrates, it is possible to induce the same cellular response in terms of spreading and stress fiber formation normally observed in cells cultured on rigid substrates. By means of a 3D magnetic twisting cytometry system, Tajik et al. have investigated the effects of planar deformations on transcription regulation, showing a loading dependency of both chromatin stretching and gene expression. They proved that cytoskeletal elements (i.e., actin and actomyosin machinery) were essential to transfer stresses from focal adhesions to the nucleus and to induce changes in transcription regulation (Tajik et al., 2016). Rysä et al. (2018) have evaluated how cyclic strains alter gene expression in neonatal rat ventricular myocytes, finding that different mechanical perturbations activate genes and transcription factors eventually involved in cardiomyocyte hypertrophic growth. Using a microfluidic device, Pagliara et al. stretched cells in the microchannel and observed nuclear auxetic behavior in the metastable transition state characterizing ESC differentiation. Surprisingly, nuclei showed a cross-sectional expansion when stretched and a cross-sectional contraction when compressed. The authors linked this auxetic phenotype to chromatin decondensation, and they hypothesized that this is directly involved in mechanotransduction processes underlying ESC commitment toward specific cellular lineages (Pagliara et al., 2014).

#### Squeezing and Shear Stress

Microfluidic devices resembling the microstructural features of cell microenviroment (i.e., pores) have been extensively used to study the mechanical processes regulating cell migration and invasion (Figure 3C). Through a device presenting different constriction widths (from 2 to 5 μm), Davidson et al. characterized nuclear deformations dynamic during cell invasion. The authors observed nuclear lamina buckling and severe intranuclear strains during cellular translocation, finding that lower Lamin A/C expression levels were associated to higher invasion potential (Davidson et al., 2015). Cell squeezing through channel constrictions has also been linked to frequent NE ruptures eventually causing DNA damage and cell death. ESCRT III machinery indeed has been found to play a central role in repairing NE avoiding cell death (both in normal and tumor cells) and then potentially contributing to both immune response and cancer progression (Denais et al., 2016; Raab et al., 2016). The effects of shear stresses on different cell functions have been evaluated by tuning fluid flow rates. Yu et al. studied the impact of shear stress on MC3T3-E1 cell proliferation and differentiation through the activity modulation of RUNX2, a key transcription factor regulating osteoblast differentiation. They observed that shear between 1.5 and 52.6 Pa promoted proliferation and osteoblast differentiation, while shear higher than 412 Pa inhibited cellular division (Yu et al., 2014). Recently, Cognart et al. have developed a microfluidic chip recapitulating some features of the blood microcirculation system to analyze how shear stress together with cell squeezing alters gene expression in breast cancer cells. They showed that these two stimuli could synergically cause important DNA damage accumulation and gene expression modification eventually leading to epithelial-to-mesenchymal transition (Cognart et al., 2020).

#### Adhesion-Mediated Forces

Besides directly applying mechanical perturbations, cell and then nuclear mechanics can also be modulated by the fine control of cell adhesion. To this aim, different substrates have been engineered with chemical, physical, or topographical cues to mimic various ECM conditions (Crowder et al., 2016; Ventre and Netti, 2016).

##### Chemical patterning

Chemical pattering is a well-established method to control cell shape (Thé et al., 2006). It relies on the insertion, on cell-repellent surfaces, of functional motifs promoting selective cell adhesion (Figure 3D). A seminal study using this approach was published in 1997 by Chen et al., who used fibronectin islands of different dimensions to study the impact of cellular shaping on cell survival. They observed that independently from the spatial distribution of adhesion processes, cellular shape *per se* was a determinant factor regulating DNA synthesis and cell apoptosis (Chen et al., 1997). Lately, Killan et al. printed rectangular and pentagonal adhesive islands to decipher the shaping effects upon MSC differentiation. They found that geometrical cues could alter cell tensional state through adhesion processes and cytoskeletal reorganization selectively leading to adipogenesis or osteogenesis (Kilian et al., 2010). With similar approach, Versaevel et al. forced cells to adhere on rectangular adhesive islands to mechanistically analyze the link between cellular and nuclear polarization. They found that changes of cell aspect ratio generate actomyosin-mediated anisotropic compressive forces acting on the nucleus that induce drastic changes on chromatin condensation and cell proliferation (Versaevel et al., 2012). Chemical patterning of adhesive molecules has been largely exploited to systematically link geometry induced cell tensional state to epigenetic responses. Many experimental evidences showed that the lone cell shape strongly affects histone acetylation, telomere dynamics, and nuclear mechanics through the modulation of the adhesion-dependent actomyosin machinery (Jain et al., 2013; Makhija et al., 2016; Roy et al., 2018). Cellular shaping has also been proposed to regulate YAP transcriptional activity, which is in turn involved in cell mechanics and focal adhesion assembly (Nardone et al., 2017). Recently, Nastaly et al. used fibronectin-coated lines to induce cell polarization, finding that cell polarity is partially transmitted to the NE and then to the nuclear interior. They also proved that Emerin plays a fundamental role in the control of nuclear polarity establishment (Nastały et al., 2020).

##### Physical cues

Cell ability to sense and readapt to mechanical/physical properties of the surrounding environment (i.e., mechanosensing) is a widely recognized and accepted concept (Engler et al., 2006; Swift et al., 2013). In particular, rigidity and rheological properties of tissues have been shown to deterministically regulate numerous biological processes in both physiological and pathological contexts (Figure 3F). Cell mechanosensing has been elegantly described by Roca-Cusachs and colleagues, who proposed a “molecular clutch model” describing the effects of material stiffness on adhesion complex formation and cytoskeletal reorganization, the determinants of both cell and nucleus tensional states (Elosegui-Artola et al., 2016). These findings inspired the development and use in cell biology research of engineered materials with controlled mechanical properties. Dupont et al. (2011) first linked mechanosensing to nuclear transcription factor activity. They discovered that substrate stiffness is sufficient to induce YAP/TAZ cytoplasmic/nuclear translocation and then its activation. Lately, using polyacrylamide gels of different stiffness, Elosegui-Artola et al. (2017) demonstrated that the YAP nuclear translocation was enabled by nucleus flattening and nuclear pore stretching/opening through cytoskeletal-mediated force transmission. By means of a high-throughput approach including the combination of biochemical and mechanical signals, Gobaa et al. (2015) established a hierarchical classification of the stimuli leading to hMSC differentiation, finding that substrate stiffness could impose specific differentiation commitments independently from biochemical cues. Recently, Yang et al. (2020) used substrates of different stiffness for the study of the molecular actors involved in cancer cell mechanosensing and progression, finding that the rigidity-independent growth generally observed in transformed cells is regulated by cytoskeletal protein depletion rather than alteration of kinases and biochemical signaling pathways. In the last years, particular attention has been devoted to the development of smart materials capable of modifying on-demand their mechanical properties mimicking the dynamic complexity of living systems (Burdick and Murphy, 2012). Guvendiren et al. developed a MeHa hydrogel-based platform changing its stiffness from 3 to 30 kPa through light-mediated stepwise crosslinking to study hMSC differentiation. Interestingly, they demonstrated that adipogenic or osteogenic differentiation was not dependent only from material stiffness, but also on how long cells were cultured on a substrate with a defined rigidity. By stiffening the substrates at different time points after cell seeding, the authors reported a time-dependent mechanical-mediated epigenetic triggering of specific differentiation pathways (Guvendiren and Burdick, 2012). Günay et al. synthetized a light-sensitive PEG-based hydrogel mimicking the dynamic rigidity variation typically observed in cardiac tissues after heart attack. This material was used to investigate the stiffness-dependent localization of NFAT, a downstream target of intracellular calcium signaling involved in the transformation of cardiac fibroblasts in myofibroblasts. Here, NFAT was shown to translocate into the nucleus only on dynamically stiffened (from 10 to 50 kPa) substrates (within 6 h), while it remained cytoplasmic in cells cultured on both 10 or 50 kPa “static” substrates, pointing out the importance of dynamic signaling in mechanotransduction (Kemal Arda et al., 2019). Beside stiffness, viscoelasticity and substrate strain energy have also been recently highlighted to affect mechanosensing. Bennet et al. extended the molecular clutch model originally developed by Roca-Cusachs et al. for separating the effects of substrate elastic and viscous components on cellular mechanoresponse. By culturing cells on lipid bilayers of different viscosities, they showed how those affect cellular properties such as cell size, focal adhesions, cytoskeletal organization, actin retrograde flow, and YAP translocation (Bennett et al., 2018). Gong et al. employed modified hyaluronic acid hydrogels to probe viscoelasticity effects on cell spreading. They showed that, on soft substrates, maximum cell spreading is observed on materials with relaxation times falling within clutch binding lifetime, while on stiff substrate, the effect of viscosity became negligible (Gong et al., 2018). Recently, Panzetta et al. analyzed the mechanical state of cells adhering on polymeric substrates displaying constant stiffness at different pre-stress levels. Results highlighted that the strain energy stored in the material affects cell mechanical state, with higher deformations leading to increased cellular stiffness. Cell–material interaction was modeled with a modified version of the classic clutch model including material pre-strain, which matched experimental outcomes (Panzetta et al., 2019).

##### Topographies

In living tissues, cells actively interact with numerous morphological cues through a process known as “contact guidance,” which dictate adhesion process formation, cytoskeletal remodeling, and then cell mechanics (Figure 3E). Understanding the basic mechanisms underpinning cell–topography interaction represents another fundamental step in both the comprehension of several biological processes and the rational design of biomaterials guiding cellular function. Indeed, the concomitant growth of materials science and fabrication technologies permitted developing micro- and nanostructured interfaces resembling basic structural elements of the ECM. Topographies act on cell behavior by influencing adhesion complex dimension as well as their orientation and distribution. Such interactions, indeed, are translated in the generation of differential cytoskeletal stresses transmitted to the nucleus eventually altering gene expression (Larsson et al., 2018; Cutiongco et al., 2020). Linear micro- and nano-topographies in the form of ridge and grooves have been shown to influence several cellular functions such as migration (Dalton et al., 2001; Pramotton et al., 2019), proliferation (Sun et al., 2016), and differentiation (Iannone et al., 2015). Here, focal adhesions align in the longitudinal direction of the pattern and their dimensions could be finely tuned even with subtle variations of the topography structural features. This, in turn, causes cell polarization along pattern direction and generates anisotropic intracellular forces dictating migration directionality and nuclear deformation (Ray et al., 2017). Among others, artificial topography has been found to be extremely effective in cell differentiation reprogramming (Dalby et al., 2014). Coez et al. fabricated microgrooved surfaces promoting the differentiation of cardiac progenitor into cardiomyocytes through an epigenetic-mediated mechanism. Notably, the differentiation efficiency was considerably higher than the one recorded *via* viral transduction (Morez et al., 2015). Similarly, Downing et al., by means of linear topographies, studied the epigenetic pathways linked to somatic cell reprogramming toward pluripotent stem cell lineage. Here, it has been shown that by tuning topography structural features, it is possible to obtain the same epigenetic modifications (i.e., histones modifications) normally induced through chemical stimulation (Song et al., 2020). Nuclear shape and mechanics could also be altered with micro pillar-based topographies. This type of topography found numerous applications in cell function regulation and in the characterization of nuclear deformability. By changing pattern dimension, indeed, these structures could induce very peculiar and cell-dependent nuclear shaping, allowing, among others, the discrimination between normal and cancer cells (Davidson et al., 2009; Badique et al., 2013; Liu et al., 2019). Micropillar topographies, through nuclear deformation, could also influence differentiation pathways as shown by Liu et al. (2016), who demonstrated a correlation between pillar height and spacing and the triggering of specific differentiation (i.e., osteogenesis or adipogenesis).

High-aspect-ratio nanostructures (nanopillars, nanoneedle, and nanowires) could imprint notable membrane and nuclear deformation and have been used in different contexts as smart tools for cytoskeletal remodeling, nuclear mechanic characterization, and cell function regulation (Crowder et al., 2016). However, except for an example of DNA damage induction, this type of structure has never been associated with gene expression modulation (Lou et al., 2018). Recently, Seong et al. showed how stem cell gene expression could be modulated by means of nanoneedle arrays. Here, lamin genes, YAP transcription, and focal adhesion gene expression were varied by changing nanoneedle distribution and structural features (Seong et al., 2020).

Since many recent studies highlighted how three-dimensional cues affect cell mechanotransduction processes differently from 2D surfaces (Pampaloni et al., 2007), in the last years, several efforts have been spent in the development of dedicated three-dimensional systems for studying cellular behavior (Nava et al., 2017; Pennacchio et al., 2018; Pennacchio et al., 2019). By means of the rolling-up technique, Koch et al. fabricated silicon-based microtubes of different diameters (from 4 to 25 μm) to assess the effects of 3D cell confinement upon nucleus integrity and growth. Here, it was found that persistent nuclear squeezing does not cause DNA damage and cell death *per se*, but can impair normal cell cycle progression leading to cell death (Koch et al., 2014). Greiner et al. (2015) embedded fibroblasts and epithelial cells into fabricated 3D microstructured scaffolds finding increased cytoplasmic and nuclear volumes compared to what is observed on 2D surfaces. Lewis et al. produced microstructured gelatin scaffolds presenting different pore sizes and recapitulating liver structural features for evaluating hepatocyte function regulation. Gene expression patterns changed in 3D environments compared to 2D, and functions like albumin secretion, CYP activity, and bile transport were found to be sensitive to pore connectivity modulation (Lewis et al., 2018). Recently Sergio et al. fabricated 3D ordered microscaffolds to assess β-catenin activity in breast cancer cell during cell invasion. The author observed that, differently from 2D substrates, the scaffold three-dimensionality could activate TCF4 transcription factor leading to β-catenin nuclear accumulation and promoting invasion of MCF-7 cells (Sergio et al., 2020).

## Conclusion

Over the past two decades, many efforts have been made to well characterize both the tight connection existing between nucleus and cytoskeleton and the nucleus intrinsic and cell-induced mechanical properties. The nucleus, indeed, is no longer considered to be the mere cage protecting cell’s genetic material and enclosing essential biological processes, including DNA replication and transcription. Instead, it is clear that structural and then mechanical properties of the nucleus largely influence various cellular functions. The observation that mutations in the Lamin gene dramatically perturb the nuclear structure leading to several diseases was the primary evidence for the importance of nuclear mechanical properties in human health. More recently, nuclear mechanics is emerging as an important factor also in cancer dissemination and probably, in the following years, we will have a clearer idea of its role in cancer onset and evolution. In this review, we introduced the basic elements to understand nuclear mechanics, the effects of its perturbation, and the most common methods employed to study it. What clearly emerged is the growing impact of advanced engineering and imaging techniques to explore and decipher the biological relevance of mechano-physical properties of the nucleus. In the future, we envisage that the further intermingling of these different disciplines will allow us to better understand the complexity of mechanotransduction.

## Author Contributions

All authors listed have made a substantial, direct and intellectual contribution to the work, and approved it for publication.

## Conflict of Interest

The authors declare that the research was conducted in the absence of any commercial or financial relationships that could be construed as a potential conflict of interest.

## Abbreviations

AFM: atomic force microscopy
CH: Calponin Homology
cPLA2: cytosolic phospholipases A2
ECM: extracellular matrix
EDMD: Emery-Dreifuss muscular dystrophy
ER: endoplasmic reticulum
FLIM: fluorescence lifetime changes
FRET: Förster resonance energy transfer
INM: inner nuclear membrane
KASH: Klarsicht/ANC-1/Syne Homology
LEM: LAP2-Emerin-MAN1
LINC: LInker of Nucleoskeleton and Cytoskeleton
LMNA: Lamin
5-LOX: 5-lopoxygenase
MAT: mesenchymal–ameboid transition
MSCs: mesenchymal stem cells
MSD: medium square displacement
NE: nuclear envelope
Nesprins: Nuclear Envelope SPectrin-Repeat proteins
NFAT: nuclear factor of activated T cells
NPCs: nuclear pore complexes
ONM: outer nuclear membrane
PL: phospholipids
PNS: perinuclear space.
